# Rapid and In-Depth Coverage of the (Phospho-)Proteome With Deep Libraries and Optimal Window Design for dia-PASEF

**DOI:** 10.1016/j.mcpro.2022.100279

**Published:** 2022-08-06

**Authors:** Patricia Skowronek, Marvin Thielert, Eugenia Voytik, Maria C. Tanzer, Fynn M. Hansen, Sander Willems, Ozge Karayel, Andreas-David Brunner, Florian Meier, Matthias Mann

**Affiliations:** 1 Department of Proteomics and Signal Transduction, Max Planck Institute of Biochemistry, Martinsried, Germany; 2 Department of Functional Proteomics, Jena University Hospital, Jena, Germany; 3 Faculty of Health Sciences, NNF Center for Protein Research, University of Copenhagen, Copenhagen, Denmark

**Keywords:** TIMS, PASEF, data-independent acquisition, phosphoproteomics, systems biology, ACN, acetonitrile, DDA, data-dependent acquisition, DIA, data-independent acquisition, EGF, epidermal growth factor, EGFR, EGF receptor, FA, formic acid, FDR, false discovery rate, GOBP, Gene Ontology Biological Process, IM, ion mobility, LC, liquid chromatography, MS, mass spectrometry, PASEF, parallel accumulation–serial fragmentation, PTM, post-translational modification, py_diAID, Python package for DIA with an automated isolation design, RT, retention time, SPD, samples per day, TIMS, trapped ion mobility spectrometry

## Abstract

Data-independent acquisition (DIA) methods have become increasingly attractive in mass spectrometry–based proteomics because they enable high data completeness and a wide dynamic range. Recently, we combined DIA with parallel accumulation–serial fragmentation (dia-PASEF) on a Bruker trapped ion mobility (IM) separated quadrupole time-of-flight mass spectrometer. This requires alignment of the IM separation with the downstream mass selective quadrupole, leading to a more complex scheme for dia-PASEF window placement compared with DIA. To achieve high data completeness and deep proteome coverage, here we employ variable isolation windows that are placed optimally depending on precursor density in the *m/z* and IM plane. This is implemented in the freely available py_diAID (Python package for DIA with an automated isolation design) package. In combination with in-depth project-specific proteomics libraries and the Evosep liquid chromatography system, we reproducibly identified over 7700 proteins in a human cancer cell line in 44 min with quadruplicate single-shot injections at high sensitivity. Even at a throughput of 100 samples per day (11 min liquid chromatography gradients), we consistently quantified more than 6000 proteins in mammalian cell lysates by injecting four replicates. We found that optimal dia-PASEF window placement facilitates in-depth phosphoproteomics with very high sensitivity, quantifying more than 35,000 phosphosites in a human cancer cell line stimulated with an epidermal growth factor in triplicate 21 min runs. This covers a substantial part of the regulated phosphoproteome with high sensitivity, opening up for extensive systems-biological studies.

## Highlights

• Optimal dia-PASEF window design with py_diAID combined with deep libraries.
• Quantification of the HeLa cell proteome to a depth of >7700 in only 44 min.
• Ion mobility–resolved phosphoproteomics determines >35,000 class I phosphosites.
• py_diAID is freely available as GUI, CLI, and Python modules.

## Graphical Abstract

**Figure:**
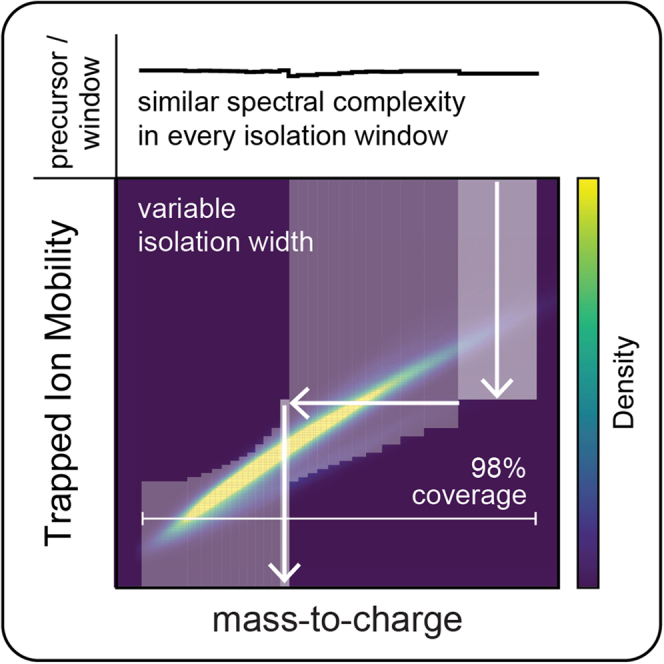

## In Brief

dia-PASEF uses the correlation of molecular weight and ion mobility in a trapped ion mobility mass spectrometer for enhanced sensitivity. However, previous methods only covered a part of the precursor space, especially for phosphopeptides. Here, we developed py_diAID for optimal placement of dia-PASEF isolation windows for nearly complete precursor coverage. Combined with short liquid chromatography gradients (100 samples per day) and project-specific in-depth libraries, we achieve substantial coverage of the proteome and phosphoproteome.

Mass spectrometry (MS)–based proteomics has become a powerful tool to study proteomes in a systematic and an unbiased manner (1). In recent years, this development has been accelerated by data-independent acquisition (DIA) (2), where predefined isolation windows cycle through the *m/z* range of interest and regularly subject the covered peptide precursors to fragmentation (3, 4, 5, 6). Although the concept of DIA was established more than a decade ago (4, 7), only the most recent DIA implementations and hardware advancements in MS and data analysis are at par or even exceeding data-dependent acquisition (DDA) with regard to sensitivity, reproducibility, and dynamic range coverage (2, 6, 8) and surpass targeted approaches in throughput and ease of use (9, 10). This holds also true for studying post-translational modifications (PTMs) (11, 12, 13).

DIA has recently shown promise in combination with trapped ion mobility (IM) spectrometry (TIMS) mass spectrometers, as demonstrated with single-cell analysis (14, 15). The TIMS tunnel is a compact and high-performance implementation of IM separation. It captures the peptides from the incoming ion beam discretizing the continuous liquid chromatography (LC) elution. Within the TIMS tunnel, each ion reaches an equilibrium position based on the opposing forces of a gas flow and an electric field gradient. Decreasing the electric field gradient elutes the peptide ions as a function of their IM (16, 17, 18, 19). In the Bruker timsTOF instruments, the TIMS device is placed upstream of mass-selective quadrupole and high-resolution time-of-flight (TOF) mass analyzer and is itself divided into two parts (20, 21, 22). The mobility separation can be synchronized with the quadrupole isolation, leading to high ion beam utilization, increased sensitivity, and decreased spectral complexity because of the additional IM dimension (6, 20, 23). This principle is termed PASEF for parallel accumulation–serial fragmentation (21, 24).

When combined with DIA (dia-PASEF), peptide precursors separate not only in the *m/z* but also in the IM dimension, in contrast to standard DIA modes (2, 6). We have observed that dia-PASEF is particularly beneficial for acquiring a wide range of proteomics data while maintaining a high sequence coverage and very high sensitivity (6, 15). Furthermore, ions are detected by inherently fast TOF analysis allowing fast DIA cycle times, which is particularly advantageous for short LC gradients (6, 25). The resulting complex spectra can be efficiently analyzed by machine learning or deep learning–based algorithms such as DIA-NN (26, 27).

Here, we set out to explore the potential of dia-PASEF to further increase coverage and quantitative accuracy on the fast and sensitive IM-MS platform. In dia-PASEF, two-dimensional precursor isolation schemes are defined in the *m/z*–IM plane. We used a Bayesian optimization algorithm ensuring optimal placement of the acquisition scheme in both dimensions. Single runs acquired with these optimal dia-PASEF methods were searched against in-depth project-specific libraries. Furthermore, we combined dia-PASEF with the Evosep One LC system, which features a preformed gradient particularly designed for high throughput by eliminating inter-run overhead (6, 28). Together, our optimized dia-PASEF workflow for high-throughput proteomics quantified more than 7000 proteins in only 21 min from quadruplicate injections of a tryptic HeLa digest.

Motivated by these proteomic results, we also investigated py_diAID (Python package for DIA with an automated isolation design) for phosphorylation analysis. On the Orbitrap MS platform, Olsen *et al.* (11) recently demonstrated an efficient combination of fast chromatography runs with DIA, quantifying more than 13,000 phosphopeptides in very short (15 min) LC–MS runs from HeLa cells using the Spectronaut software (Biognosys AG). In a small-scale study, Ishihama *et al.* (29) showed that phosphopeptide analysis benefits from the additional IM dimension in PASEF. For large-scale PTM studies, our optimized py_diAID acquisition schemes cover nearly all theoretical phosphopeptide precursors and quantified expected changes in the well-studied epidermal growth factor (EGF) receptor (EGFR) signaling pathway with minimal time and sample consumption.

## Experimental Procedures

### Experimental Design and Statistical Rationale

All experiments were done using HeLa cell lysate obtained from HeLa S3 cells (American Type Culture Collection) and routinely used for proteomics method development and benchmark experiments (supplemental Fig. S1). Altogether, the dataset includes 322 raw data files (uploaded to PRIDE, see later). We used the same HeLa batch for generating libraries and single-run data of both proteome and phosphoproteome measurements. In brief, proteome measurements with different gradient lengths and the technical comparisons of the original and optimal dia-PASEF methods for phosphoproteomics were acquired in quadruplicates. Unless otherwise mentioned, 200 ng HeLa lysate was used for single-run proteome and 100 μg for the single-run phosphopeptide enrichment experiments. The libraries were acquired as described later. The experimental design and statistical rationale are described in the respective figure legends. The EGF experiment was performed in biological triplicates to determine significantly different phosphosite levels between the EGF-treated and control samples. Technical quadruplicates were acquired to evaluate reproducibility and quantitative accuracy by calculating CVs and mean of the replicate injections. Moreover, we alternated the MS run order to avoid potential carryover effects or any similar biases.

### Sample Preparation

HeLa S3 cells (American Type Culture Collection) were cultured in Dulbecco’s modified Eagle’s medium (Life Technologies Ltd) containing 20 mM glutamine, 10% fetal bovine serum, and 1% penicillin–streptomycin. Sample preparation was essentially performed as previously described in the in-stage tip protocol (30). In brief, the cells were washed with PBS and lysed. Protein reduction and alkylation and digestion with trypsin (Sigma–Aldrich) and LysC (WAKO) (1:100 dilution, enzyme/protein, w/w) were performed in one step. Resulting peptides were dried and reconstituted in a solution A∗ (0.1% TFA/2% acetonitrile [ACN]). Peptide concentrations were measured optically at 280 nm (Nanodrop 2000; Thermo Fisher Scientific), and 200 ng peptides were loaded onto Evotips for LC–MS/MS analysis as described previously (15). The Evotips were washed with 0.1% formic acid (FA)/99.9% ACN, equilibrated with 0.1% FA, loaded with the sample dissolved in 0.1% FA, and washed with 0.1% FA.

For phosphoproteomics, HeLa cells at a plate confluence of 80% were treated for 10 min with 100 ng/ml animal-free recombinant human EGF (PeproTech) or Gibco distilled water (Thermo Fisher Scientific) and washed three times with ice-cold TBS before lysis in 2% sodium deoxycholate in 100 mM Tris–HCl (pH 8.5) at 95 °C. Protein concentrations were determined using the bicinchoninic acid assay, and samples were then reduced and alkylated with 10 mM Tris(2-carboxyethyl)phosphine and 40 mM chloroacetamide, respectively. Altogether, 25 mg protein material of sample was used for the library generation, 8 mg for EGF-treated experiments including method benchmarking, and 4 mg for untreated experiments. The sample was digested with trypsin (Sigma–Aldrich) and LysC (WAKO) (1:100 dilution, enzyme/protein, w/w) overnight and subsequently desalted using Sepax Extraction columns (Generik DBX). Each cartridge was prepared with 100% MeOH and 99% MeOH/1% TFA. After equilibration with 0.2% TFA, the samples were loaded with a protein concentration of 1 mg/ml, washed with 99% isopropylamine/1% TFA, 0.2% TFA/5% ACN, and 0.2% TFA solutions. The peptides were eluted with 5% NH_4_OH/80% ACN. Lyophilized peptides were reconstituted in equilibration solution (1% TFA/80% ACN), and 100 μg peptide material per sample/AssayMAP cartridge, each containing 5 μl Fe(III)–nitrilotriacetic acid, was enriched for phosphopeptide with the AssayMAP bravo robot (Agilent) (31). Phosphopeptides were dried in a SpeedVac for 20 min at 45 °C and loaded onto Evotips as described previously.

### High-pH Reverse-Phase Fractionation for Library Generation

To generate proteome libraries, 10 and 60 μg peptides were separated with high-pH reverse-phase chromatography into 24 and 48 fractions, respectively, on a 30 cm C_18_ column with an inner diameter of 250 μm at a flow rate of 2 μl/min using the spider sample fractionator (32). The gradient consisted of the binary buffer system (PreOmics GmbH). The buffer B concentration of 3% was increased to 30% in 45 min, 40% in 12 min, 60% in 5 min, and 95% in 10 min. After washing at 95% for 10 min, buffer B concentration was re-equilibrated to 3% in 10 min. The exit valve concatenated the eluted peptides automatically by switching after a defined collection time (80 s for 24 and 60 s for 48 fractions). The fractions were dried in a SpeedVac and reconstituted in solution A∗. A quarter of each fraction was loaded onto Evotips for LC–MS/MS analysis. Later, we will refer to “the reference proteome library” that represents a 24 high-pH fraction and DDA–PASEF spectral library of a tryptic HeLa digest acquired with a 21 min Evosep gradient.

To generate a phosphoproteome library, peptides obtained from the EGF-stimulated cells were separated using an UFLC system (Shimadzu). About 6 mg peptide material was fractionated with a binary buffer system: A (2.5 mM ammonium bicarbonate) and B (2.5 mM ammonium bicarbonate/80% ACN). The peptides were loaded onto a reversed-phase column (ZORBAX 300Extend-C_18_; Agilent) and separated at a 1 ml/min flow rate at 40 °C. The buffer B concentration of 2.5% was increased to 38% in 82.5 min, 75% in 2 min, and 100% in 8 min. It stayed at 100% for 2 min and was reduced to 2.5% in 2 min. In total, 95 fractions were collected, and fractions with low peptide yield, as determined using Nanodrop, were pooled (supplemental Table S1) and dried in a SpeedVac. Next, 76 fractions were enriched for phosphopeptide, which were subsequently loaded onto Evotips.

### LC–MS/MS Analysis

The Evosep One LC system coupled with a timsTOF Pro mass spectrometer (Bruker) was used to measure all samples. The 60 and 100 SPD (samples per day) methods required an 8 cm × 150 μm reverse-phase column packed with 1.5 μm C_18_-beads (PepSep), and the 30 SPD method a 15 cm × 150 μm column with 1.9 μm C_18_-beads (PepSep) at 40 °C. The analytical columns were connected with a fused silica ID emitter (10 μm ID; Bruker Daltonics) inside a nanoelectrospray ion source (Captive spray source; Bruker). The mobile phases comprised 0.1% FA as solution A and 0.1% FA/99.9% ACN as solution B.

The library samples were acquired in DDA–PASEF mode with four PASEF/MSMS scans at a throughput of 60 and 100 SPDs and 10 PASEF/MSMS scans at 30 SPD per topN acquisition cycle. Singly charged precursors were filtered out by their position in the *m/z*–IM plane, and only precursor signals over an intensity threshold of 2500 arbitrary units were picked for fragmentation. While precursors over the target value of 20,000 arbitrary units were dynamically excluded for 0.4 min, ones below 700 Da were isolated with a 2 Th window and ones above with 3 Th. All spectra were acquired within an *m/z* range of 100 to 1700 and an IM range from 1.51 to 0.6 V cm^−2^.

We described the original dia-PASEF method in the study by Meier *et al.* (6). The dia-PASEF methods optimized here with py_diAID cover an *m/z* range from 300 to 1200 for proteome and from 400 to 1400 for phosphoproteome measurements. Each method includes two IM windows per dia-PASEF scan with variable isolation window widths adjusted to the precursor densities. Eight, 12, and 25 dia-PASEF scans were deployed at a throughput of 100 (cycle time: 1.0 s), 60 (cycle time: 1.4 s), and 30 SPDs (cycle time: 2.7 s), respectively. We created dia-PASEF methods with equidistant window widths (supplemental Fig. S5) with the software “Compass DataAnalysis” (Bruker Daltonics). These acquisition schemes are plotted on top on a kernel density estimation of precursors from a reference library in supplemental Figs. S2–S4. The IM range was set to 1.5 and 0.6 V cm^−2^. The accumulation and ramp times were specified as 100 ms for all experiments. As a result, each MS1 scan and each MS2/dia-PASEF scan last 100 ms plus additional transfer time, and a dia-PASEF method with 12 dia-PASEF scans has a cycle time of 1.38 s. The collision energy was decreased as a function of the IM from 59 eV at 1/*K*_0_ = 1.6 V cm^−2^ to 20 eV at 1/*K*_0_ = 0.6 V cm^−2^, and the IM dimension was calibrated with three Agilent ESI Tuning Mix ions (*m/z*, 1/*K*_0_: 622.02, 0.98 V cm^−2^, 922.01, 1.19 V cm^−2^, 1221.99, and 1.38 V cm^−2^). For phosphoproteomics experiments, the collision energy was decreased from 60 eV at 1.5 Vs cm^−2^ to 54 eV at 1.17 Vs cm^−2^ to 25 eV at 0.85 Vs cm^−2^ and end at 20 eV at 0.6 Vs cm^−2^.

### Raw Data Analysis

We employed DIA-NN, MSFragger, and Spectronaut for transforming raw data into precursor and fragment identifications based on 3D peak position (retention time [RT], *m/z* precursor, and IM). In each case, all data were searched against the reviewed human proteome (UniProt, November 2021, 20,360 entries without isoforms) with trypsin/LysC as digestion enzymes. Cysteine carbamidomethylation was set as fixed modification. Methionine oxidation, methionine excision at the N terminus, and in the case of the phosphoproteome searches, phosphorylation (STY) was selected as variable modifications. A maximum of two missed cleavages and up to three variable modifications were allowed.

The project-specific libraries for DIA-NN analyses were generated with FragPipe (27) (FragPipe 16.2, MSFragger 3.4 (33, 34, 35), Philosopher 4.0.0 (36), Python 3.8, EasyPQP 0.1.25 (https://github.com/grosenberger/easypqp)). The default settings were kept except that the precursor mass tolerance was set from −20 to 20 ppm and the fragment mass tolerance to 20 ppm. In addition, Pyro-Glu or ammonia loss at the peptide N terminus and water loss on N-terminal glutamic acid were selected as variable modification. The output tables were filtered for a 1% false discovery rate (FDR) using the Percolator (37, 38) and ProteinProphet (39) option in FragPipe (supplemental Table S2).

DIA-NN 1.8 was used to analyze the single-shot experiments against the project-specific libraries generated with FragPipe (27). The default settings were kept except that we changed the charge state to 2 to 4. The precursor’s *m/z* range was restricted from 300 to 1200 for proteome and 400 to 1400 for phosphoproteome analysis. The fragment *m/z* range was set from 100 to 1700, and the mass and MS1 accuracy was set to 15 ppm. “Match between run” was enabled, whereas “protein inference” was disabled. We also enabled “robust LC (high precision)” as the quantification strategy. The proteomics output tables were filtered for a maximum of 1% of *q* value at both precursor and global protein levels. For phosphoproteomics, the PTM *q* value also had to be a maximum of 1%. The “PG.MaxLFQ” column integrated in the DIA-NN output tables reports normalized quantity employing the MaxLFQ principle (40) and was used for quantitative analysis on the protein level. For our phosphoproteomics analysis, we used the scoring of post-translational sites implemented in DIA-NN with “PTM.Site.Confidence” indicating the localization probability (13).

Spectronaut (version 16) (3) was used for comparative analysis, and we used the same search settings as described previously if not stated differently. The FDR cutoff was set to 1%. The precursor peptide and *q* value cutoffs were 0.2 and 0.01, respectively. The protein *q* value experiment and run wide cutoffs were 0.01 and 0.05, respectively. The dataset was analyzed with a sparse *q* value, and no imputation was performed. For phosphoproteomics experiments, the PTM localization cutoff was set to 0. The results were filtered for the best N fragments per peptide between 3 and 25.

Peptide collapse (version 1.4.1), a plug-in tool for Perseus (41), collapsed peptide output tables from DIA-NN or Spectronaut to phosphosite tables using default settings and a localization cutoff of 0.75 (class I sites) (11). The DIA-NN output table was reformatted by renaming all columns and entries calculating peptide positions to conform to the format required for the plug-in tool. For collapsing, Perseus took only phosphorylation into account. During collapsing phosphopeptide ions to phosphosites, each phosphosite corresponding to the same peptide obtains the same intensity; however, imputation may lead to differences in fold changes. If the same phosphosite was identified on different peptides, which may also have modifications other than phosphorylation or different charge states, the intensities were summed up.

### Statistical Analysis

Visualization and statistical analyses were performed using the output tables of DIA-NN or Spectronaut with Python (version 3.8, Jupyter Notebook; Project Jupyter) and the packages pandas (1.4.2) and pyfaidx (0.6.1) for data accession and py_diAID (0.0.16), AlphaMap (0.1.10), matplotlib (3.4.3), and seaborn (0.11.2) for visualization. The statistical analysis of the EGF experiment was performed in Perseus (version 1.6.2.2). Log_2_-transformed intensities were filtered for 100% valid values in at least one condition. The missing values were replaced drawing from a normal distribution (width 0.3 and downshift 1.8). Next, we applied the two-sided Student’s *t* test (S_0_ = 0.1, FDR = 0.05) to obtain the significantly changing phosphorylated peptides. A Fisher’s exact test was performed for Gene Ontology term and Kyoto Encyclopedia of Genes and Genomes pathway enrichment analysis (*p* < 0.002).

## Results

### Principle and Limitations of the Original dia-PASEF Window Design

**Fig. 1:**
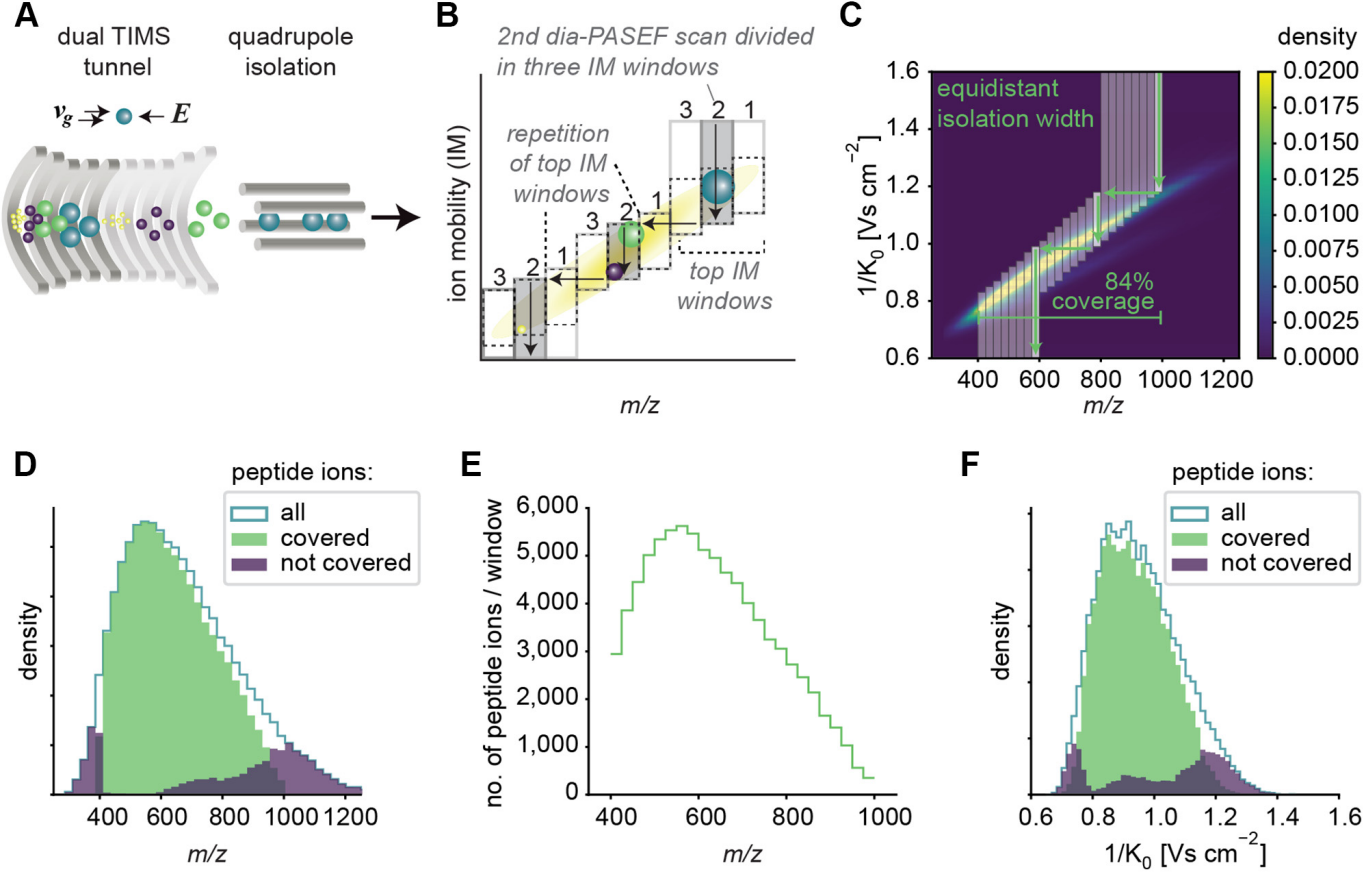
**Principle of dia-PASEF on a timsTOF with equidistant two-dimensional isolation windows.***A*, schematic of a TIMS tunnel followed by quadrupole isolation. *B*, dia-PASEF acquisition scheme depicting three dia-PASEF scans divided into three ion mobility (IM) windows. *Vertical arrows* indicate the elution of the ions with decreasing electrical field, and *horizontal arrows* indicate the movement of the quadrupole. The pattern of the *top* IM windows is repeated, and the *top* and *bottom* IM windows are extended to the upper and lower IM range, respectively. *C*, original dia-PASEF acquisition scheme (6) plotted on a kernel density distribution of all precursors. One dia-PASEF scan is divided into three IM windows by three distinct movements of quadrupole isolation. This scheme comprises eight dia-PASEF scans with equidistant isolation width covering in total 84% of the peptide ion population. *D*, histogram of *m/z* of all peptides covered by the acquisition method in (*C*), and peptides not covered by the method but identified in a separately recorded spectral library. *E*, number of peptide ions per isolation window. *F*, histogram of IMs of all peptides covered by the acquisition method, and peptides not covered by the method but identified in a separately recorded spectral library. The subfigures *C*–*F* are based on a reference proteome library (see the Experimental Procedures section). DIA, data-independent acquisition; PASEF, parallel accumulation–serial fragmentation; TIMS, trapped ion mobility spectrometry.

In the timsTOF mass spectrometer (Bruker Daltonics), a dual TIMS tunnel releases the captured peptide ion species individually as a function of their mobility. In a PASEF MS–MS scan, a quadrupole transmits part of the ion beam where the precursor *m/z* values fall into a predefined isolation window (Fig. 1*A*). These precursors are subsequently fragmented by applying a particular collision energy. A downstream TOF analyzer acquires high-resolution mass spectra. In dia-PASEF, changing the quadrupole position is synchronized to the IM elution, increasing the MS efficiency because the isolation window is placed on top of the precursor cloud (6). This movement happens in distinct steps and thereby divides one PASEF scan into multiple IM windows. The quadrupole isolation window is first placed at high *m/z* for a certain amount of time, after which it jumps to a position in the lower *m/z* range. This transition point corresponds to a particular IM value for each dia-PASEF scan. In each subsequent dia-PASEF scan, the starting *m/z* window is offset to lower values (Fig. 1, *B* and *C*). Together, these isolation windows cover a large proportion of the *m/z* and the IM dimensions, constituting a two-dimensional acquisition scheme (Fig. 1*B*).

Because of software constraints, the original dia-PASEF methods (6) comprise a repeating pattern of the top IM windows per dia-PASEF scan. This leads to a configuration with equidistant quadrupole isolation widths (Fig. 1*B*). As a result, covering a wide *m/z* range comes at the cost of a high cycle time and reduced quantitative accuracy because of lower elution peak coverage. Alternatively, many peptide ions outside the *m/z* range would not be included in the acquisition scheme (Fig. 1, *C* and *D*).

Moreover, when using equidistant isolation windows, the distribution of peptide ions per window is imbalanced, resulting in a high spectral complexity in highly dense regions (Fig. 1*E*). Finally, this scheme for acquisition window setting is also suboptimal in the IM dimension (Fig. 1*F*).

### Establishing an Optimal dia-PASEF Window Design

We first investigated the optimal balance between the number of dia-PASEF scans and IM windows per dia-PASEF scan to obtain a deep proteome coverage and quantitative accuracy. As described previously, the original dia-PASEF method included three IM windows per dia-PASEF scan. Having more IM windows per dia-PASEF scan reduces cycle time but also diminishes precursor coverage because of smaller isolation windows in the IM dimension (supplemental Fig. S5, *A* and *B*). For instance, splitting the isolation width into two parts halved the complexity per spectrum and thereby increased identifications. However, doubling the number of dia-PASEF scans increases cycle time, which worsens the quantitative accuracy since only half as many data points are collected over one elution peak (supplemental Fig. S5*A*). We tested the impact of increasing the number of IM windows per dia-PASEF scan and found that two IM windows per dia-PASEF scan are optimal (supplemental Fig. S5*C*). While optimizing the cycle time of DIA methods, one has to prioritize for the desired trade-off between identification and quantitative accuracy. The Olsen and Reiter groups achieving an average of for four and eight data points per peak, respectively (11, 42), and here we aimed at six points per elution peak. In the case of 21 min gradients (60 SPD), we empirically found an average peak width of 8.3 s (base to base, as reported by DIA-NN, see supplemental Fig. S5*D*). Each individual dia-PASEF scan takes around 100 ms plus one 100 ms MS1 scan per cycle and overhead time. Hence, 12 dia-PASEF scans amount to a cycle time of 1.4 s, representing an optimal trade-off between the adequate quantitative representation of the LC elution peak and proteomics depth (see the Experimental Procedures section, supplemental Fig. S5*E*). If a study requires a stronger focus on quantitative accuracy, a lower number of dia-PASEF scans and hence shorter cycle time may be beneficial.

**Fig. 2:**
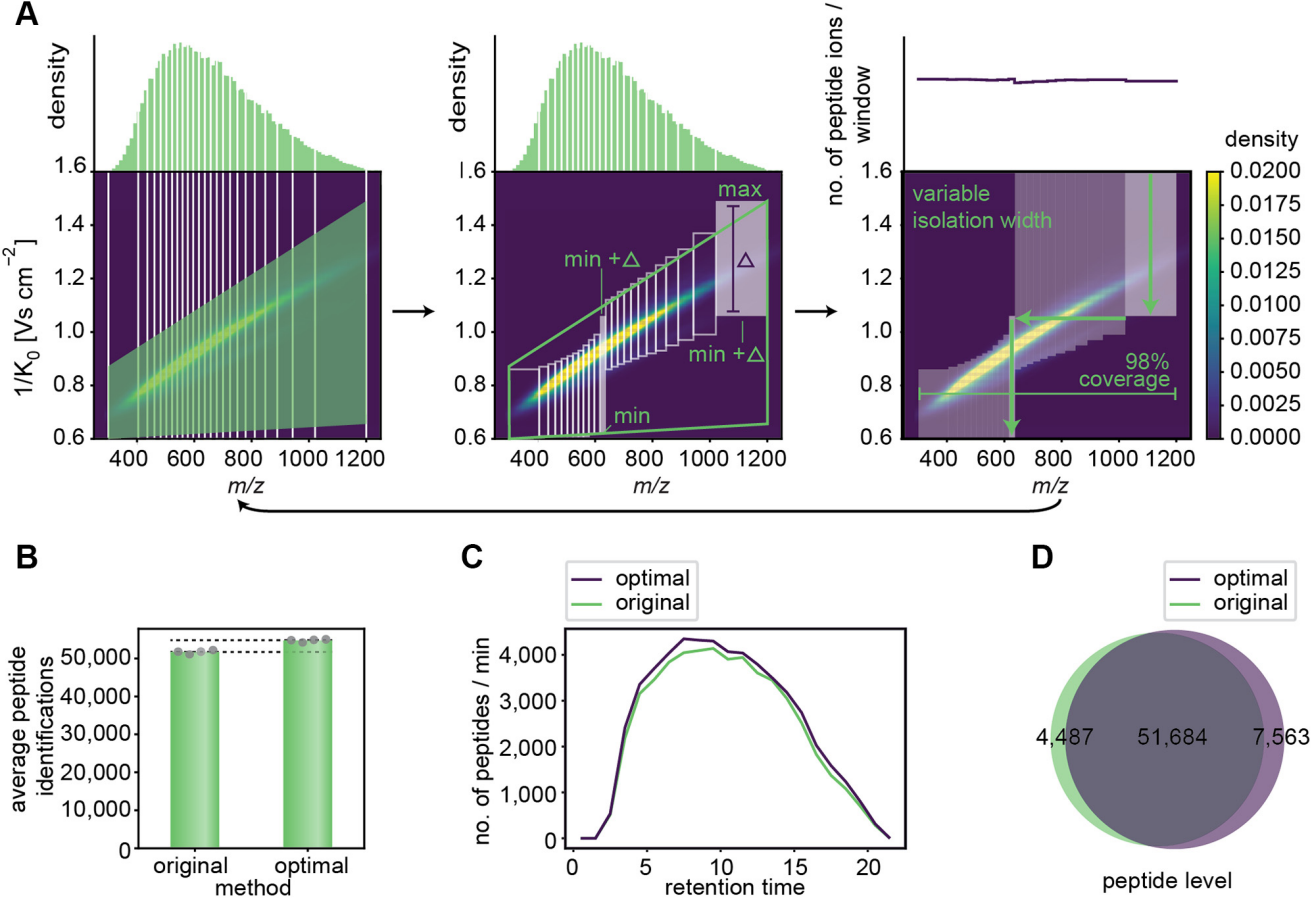
**py_diAID algorithm and evaluation.***A*, py_diAID design of the optimal acquisition scheme and window placement for a 21 min gradient (60 SPD, Evosep) with variable widths to balance the distribution of peptide ions, providing nearly complete peptide ion coverage. The *left panel* illustrates the first steps of the py_diAID algorithm: defining the *m/z* range of interest, binning the peptide ions in the *m/z* dimension and definition of the scan area in the IM dimension. *Middle panel*, calculation of the isolation window dimensions and coordinates based on the scan area. *Right panel*, extension of the isolation windows to the limits of the IM ranges. The *arrow* at the *bottom* indicates that the py_diAID algorithm evaluates the new acquisition scheme, defines the following test set of scan area parameters by Bayesian optimization, and resumes with the steps in the *left panel*. This is repeated for a user-defined number of iterations (more details in supplemental Fig. S6). *A* is plotted on top of a kernel density distribution based on the reference proteome library. *B*, average peptide identifications by the original and optimal dia-PASEF methods. *C*, number of peptides identified per minute over the entire retention time. *D*, Venn diagram showing the shared and unique peptides identified by both methods. Data in *B*–*D* are from quadruplicate injections of 200 ng tryptic HeLa digest with a 21 min gradient and analyzed with the reference proteome library. DIA, data-independent acquisition; IM, ion mobility; PASEF, parallel accumulation–serial fragmentation; py_diAID, Python package for DIA with an automated isolation design; SPD, samples per day.

Given the limitations of our previous two-dimensional acquisition scheme, we needed to place and adjust *m/z* and IM isolation windows flexibly. Existing tools such as “Define dia-PASEF Region” in Compass DataAnalysis (Bruker) or the “dia-PASEF window Editor” in TimsControl (Bruker) require the manual fitting of the scan area onto the peptide ion population and only generate isolation windows with equidistant widths. Therefore, we developed py_diAID. It places two-dimensional dia-PASEF acquisition schemes in the *m/z*–IM plane based on desired parameters (number of dia-PASEF scans, covered *m/z* and IM range, and cycle time) and the empirical acquired reference data, which can be a proteomics library containing precursor ion information. The algorithms in py_diAID optimally adjust the variable quadrupole isolation widths according to the precursor density, aiming for an equal number of precursors fragmented per isolation window. Our simulations show that variable isolation widths enable short acquisition cycles covering essentially the entire *m/z*–IM range (Fig. 2*A*, *right panel*).

Our algorithm first bins the precursor ion populations equally along the *m/z* dimension. A trapezoid defines the extent of scan area and the position of the acquisition scheme in the *m/z*–IM plane (Fig. 2*A*, *left panel*). Based on this, py_diAID calculates the optimal dimensions of each isolation window (Fig. 2*A*, *middle panel*) and extends the top and bottom IM windows to the limits of the measured IM range to maximize the covered peptide ion population (Fig. 2*A*, *right panel* and supplemental Fig. S6). The selected mass window of the quadrupole jumps at the determined transition point of each IM window within each dia-PASEF scan. In each subsequent dia-PASEF scan, the starting *m/z* window is offset to lower values based on the individual width of the previous window (Fig. 2*A*). Next, py_diAID evaluates the generated acquisition scheme based on the covered precursor ions of an experimentally acquired library or subset thereof, for example one filtered by a charge state or by a population of modified peptides. This is a multivariant nonlinear optimization problem, and we used the gp_minimize module provided by the Scikit-Optimize (skopt) library in Python to perform this task that is highly used in machine and deep learning for the hyperparameter optimization (see the Experimental Procedures section). Its inputs are the trapezoid corners, and it iteratively decides which parameters should be tested next based on the aforementioned evaluation. This process is repeated for many iterations (about 200 in practice, supplemental Fig. S7) until it converges to the best window placement. py_diAID is available as a Python module, a command-line interface, and a graphical user interface on all major operating systems under an Apache 2.0 license (supplemental Fig. S8). The source code is freely available on GitHub (https://github.com/MannLabs/pydiAID).

We first benchmarked the optimal dia-PASEF methods designed with py_diAID against the original dia-PASEF method, which we termed “high speed” in our original dia-PASEF publication (6). That method covered 88% of all doubly and 71% of all triply charged precursors in the “reference library,” which was generated with FragPipe. In contrast, the optimal dia-PASEF method calculated by py_diAID reached 99% and 94%, respectively. The original dia-PASEF method had already been extensively and manually optimized for the short gradient lengths and the tryptic HeLa digest employed here. This explains why the number of experimentally identified proteins is very similar between both methods (supplemental Table S3). However, even in this case, the optimal acquisition scheme of py_diAID increased the number of identified peptides by 6% in single-run injections (Fig. 2*B*) and across the entire RT (Fig. 2*C*), whereas the number of peptide identifications in replicate injections deviates only by 1%. Inspection of the data shows that the additional peptides originate both from the previously not covered regions and from the most dense elution times. More than 80% of all identified peptides were commonly identified by both methods (Fig. 2*D*). In other applications, such as phosphoproteomics, the gains by py_diAID were much larger (see below).

**Fig. 3:**
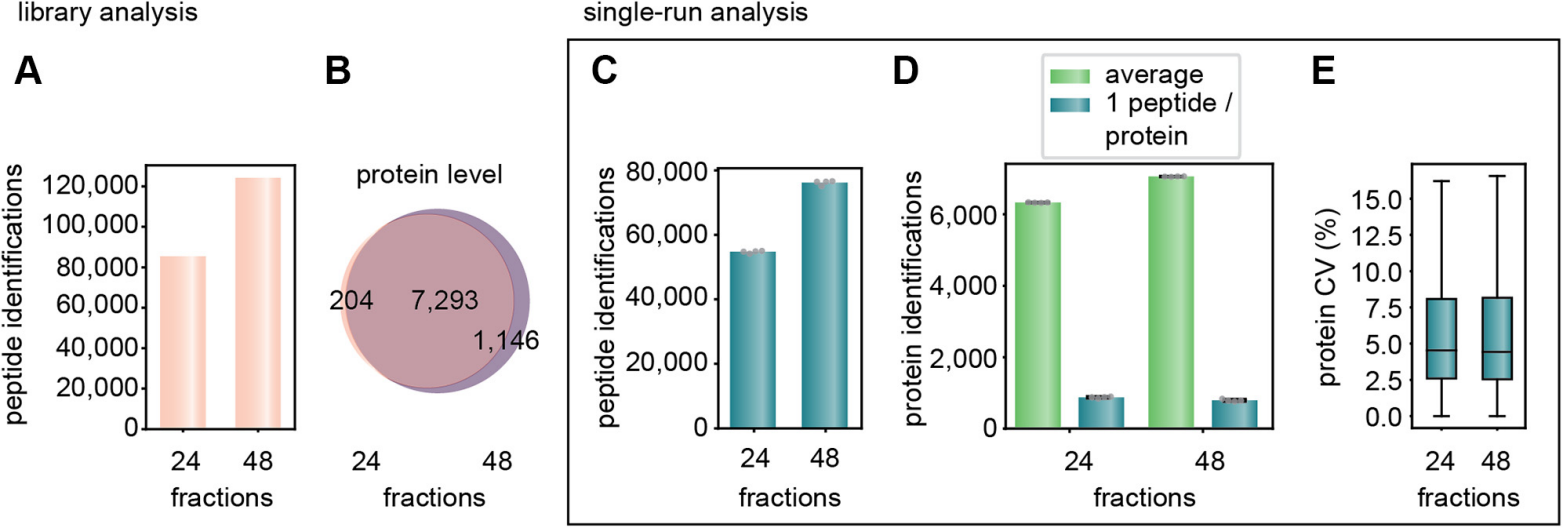
**Workflow optimization for the 21 min gradient with project-specific deep libraries.***A*, peptides identified of the reference *versus* the project-specific deep library for 21 min runs. *B*, shared proteins and depth on the protein level in the two libraries. *C*, average peptide identification of four single-run injections. These data and the one in (*D*) and (*E*) were generated from quadruplicate injections of 200 ng tryptic HeLa digest acquired with a 21 min gradient and searched with the reference (24 fractions) or project-specific library (48 fractions). *D*, average protein identifications and identifications with only one peptide in the single runs. *E*, CVs at the protein level based on the MaxLFQ algorithm of DIA-NN. Boxplots show the median (*center line*), 25th, and 75th percentiles (*lower* and *upper box limits*, respectively), and the 1.5× interquartile range (*whiskers*). n = 6384 (24 fractions) and 7121 (48 fractions) shown in *C*. DIA, data-independent acquisition.

**Fig. 4:**
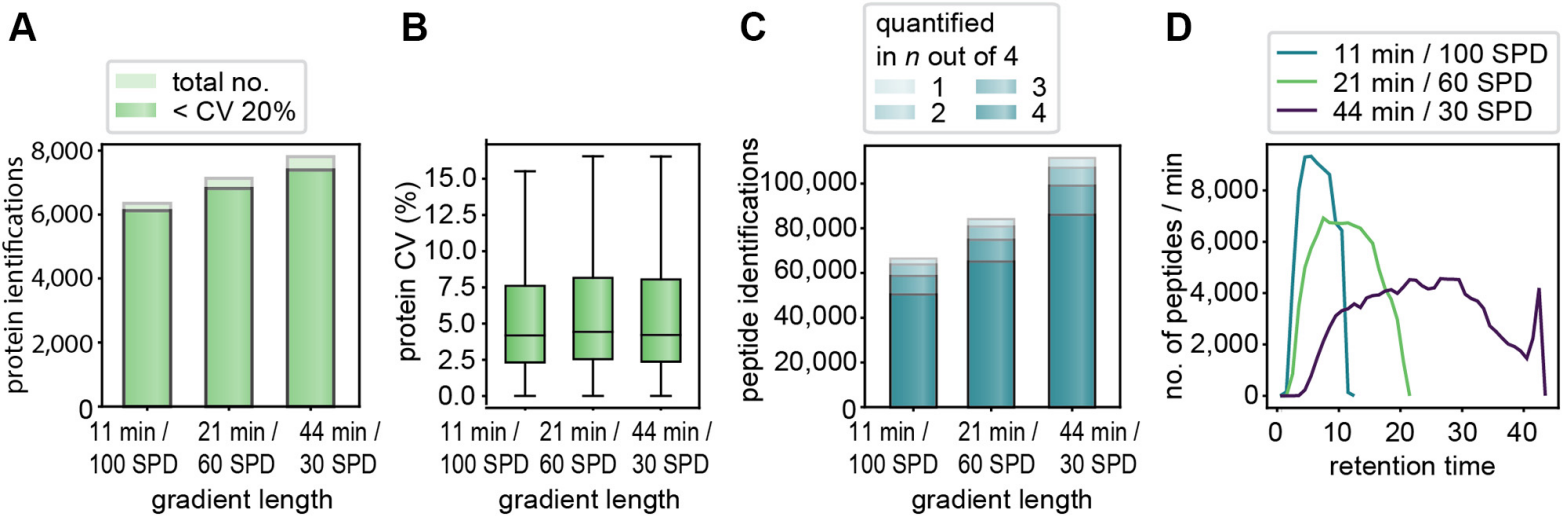
**Comparison of different gradient lengths/throughput based on single-run analysis.***A*, all single-run identifications and those with a CV <20% for the 11, 21, and 44 min gradients. *B*, CVs at the protein level based on the MaxLFQ algorithm of DIA-NN. Boxplots show the median (*center line*), 25th and 75th percentiles (*lower* and *upper box limits*, respectively), and the 1.5× interquartile range (*whiskers*). n = 6341 (11 min/100 SPD) and 7121 (21 min/60 SPD), and 7802 (44 min/30 SPD) shown in panel *A*. *C*, analysis of peptide quantification in n out of four technical replicates shows that the large majority is quantified consistently. *D*, the number of peptides per second over the retention time for the three gradient lengths. The data were acquired in quadruplicate injections of 200 ng HeLa digest and analyzed with 48 fraction, DDA–PASEF libraries each recorded with the corresponding gradient length. 11-min library: 8553 proteins and 122,105 peptides; 21-min library: 8439 proteins and 124,155 peptides; 44-min library: 9461 proteins and 175,839 peptides. DDA, data-dependent acquisition; DIA, data-independent acquisition; PASEF, parallel accumulation–serial fragmentation.

**Fig. 5:**
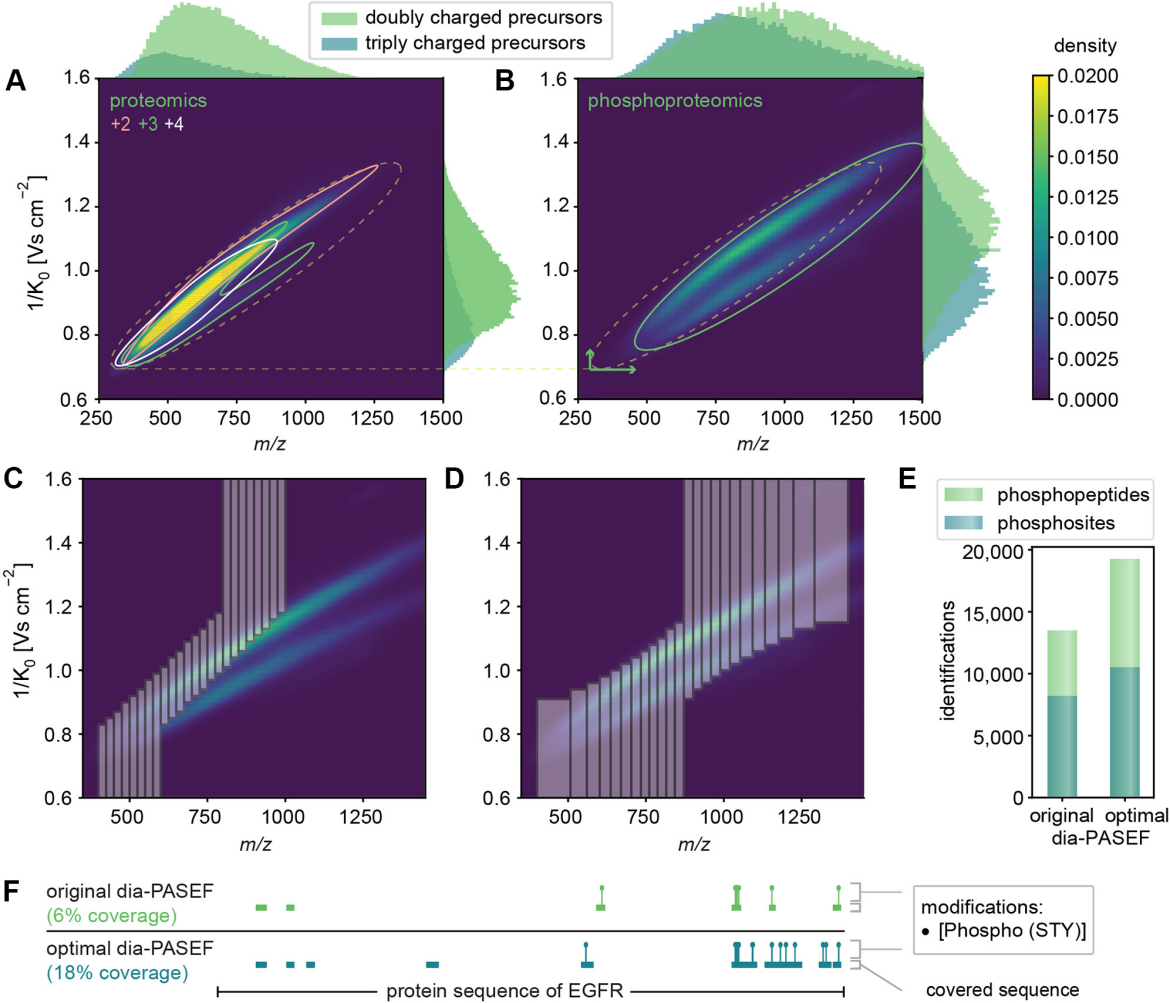
**Method optimization specifically for phosphoproteomics.***A*, peptide distribution of a proteomics digest displayed as kernel density estimation dependent on the charge and histograms of the abundance of differently charged precursors based on our deep proteomics library. *B*, peptide distribution of a phosphoproteomics digest displayed as kernel density estimation and histograms of the abundance of differently charged precursors based on our phosphopeptide library. *C*, original dia-PASEF method plotted on top of the phosphopeptide library. *D*, optimal dia-PASEF method tailored to the phospholibrary. *E*, identified phosphosites and phosphopeptides based on quadruplicates of 100 μg EGF-stimulated and enriched HeLa digest, separated within 21 min, and searched with DIA-NN against the phospholibrary. *F*, AlphaMap visualization (47): Protein sequence coverage of the EGF receptor (EGFR) depending on the acquisition method. DIA, data-independent acquisition; EGF, epidermal growth factor; PASEF, parallel accumulation–serial fragmentation.

### Deep Proteome Coverage in Short LC Gradients

We next investigated if coupling our optimized dia-PASEF methods with project-specific, in-depth libraries yields higher peptide identification and improves quantification accuracy. To generate such an in-depth library, we separated 15 μg of the HeLa sample that we also used for single dia-PASEF acquisitions into 48 concatenated fractions using high-pH reverse-phase chromatography of the Spider fractionator (see the Experimental Procedures section) (32). These fractions were measured in DDA–PASEF mode and again analyzed with FragPipe and its SpecLib workflow. We compared our “reference library” generated with limited sample amount (2.5 μg proteolytic digest) and 24 fractions to the new one with ample sample amount (15 μg) and twice as many fractions. As expected, the latter was substantially larger, containing 45% more peptides (counting all modifications) and 13% more proteins. Altogether, this deep library constructed from 21 min runs comprised 124,155 peptides and 8439 different protein groups (Fig. 3, *A* and *B*).

Next, we compared single dia-PASEF runs with reference *versus* deep library using DIA-NN and found a corresponding increase in the proteome depth (39% more peptides and 12% more proteins) (Fig. 3, *C* and *D*). Using the deep library identified 76,214 ± 1021 peptides and the reference library 51,711 ± 641 peptides (Fig. 3*C*). With the deep library, an astounding 7056 ± 8 proteins were identified with our optimized acquisition scheme in each of four replicate runs on average. Specifically, with the reference library, DIA-NN reported 14% significant protein identifications on the basis of one peptide, and this percentage decreased slightly to 11% with the deeper library (Fig. 3*D*).

Quantitative reproducibility between the quadruplicates was virtually identical when using the reference or deep library (4.5% *versus* 4.4% on protein level and 12.1% *versus* 13.45% on peptide level) (Fig. 3*E*). Taken together, we found that single-run identification benefited from a project-specific in-depth library while maintaining the accuracy of quantification. We therefore used the library of 48 fractions for all 21 min runs to generate equivalent libraries for evaluating a range of gradient lengths as described next (referred to as “project-specific deep libraries”).

We next investigated the effect of even shorter gradients as well as somewhat longer gradients on proteome depths and quantitative accuracy. As before, each library was acquired with DDA–PASEF and 15 μg HeLa lysate separated into 48 fractions. Extending the gradient to 44 min (30 SPD method on the Evosep One system) identified an average of 7756 ± 6 proteins based on 100,900 ± 634 peptides (including all modifications). This represents an identification increase of 10% on protein level in comparison to the 21 min gradient. The median CV between the quadruplicates was 4% at the protein level for these technical replicates, and 7393 protein groups had CVs below 20% (Fig. 4, *A* and *C*).

We expected that the fast scan rate of the timsTOF, together with our optimized method, might still accurately measure a large part of the proteome even in very short gradients (6, 27). Indeed, the 100 SPD method (11 min gradient) still identified 6285 ± 18 proteins (59,811 ± 368 peptides). Quantitative accuracy reported by DIA-NN did not suffer and remained at a median CV of 4%. Taking only the proteins with CVs equal or below 20%, the 100 SPD method still resulted in 6121 proteins, covering 83% of proteins that could be accurately quantified with the 44 min gradient while substantially reducing the analysis time (Fig. 4*A*). Rank order reproducibility was also high for these technical replicates for all gradient lengths (supplemental Figs. S9 and S10, *r* = 0.999 for proteins and *r* = 0.992 for peptides). As expected, the number of peptides identified per minute decreased when increasing the gradient length, whereas the 11-min gradient reached the highest numbers (9330 peptides per minute translating to 155 peptide identifications per second at the apex, Fig. 4*D*).

In conclusion, our data show that our improved workflow constitutes a powerful technological platform capable of accurately quantifying a large part of the proteome at high throughput.

### Comparison of Proteome Results Between DIA-NN and Spectronaut

The aforementioned analyses were all performed with the DIA-NN package. To determine if our results depend on the software used, we employed Spectronaut (3), another widely used software package (11, 43). This revealed that both packages identified comparable numbers of proteins. For instance, in the 60 SPD method, Spectronaut reported 7285 significant protein groups, whereas DIA-NN reported 7056 significant protein groups (supplemental Fig. S11*A*). In the version tested (Spectronaut 16), this also held for even shorter gradients (6250 *versus* 6285).

Having established that the overall protein numbers are similar, we next investigated the overlap between the found proteins. As DIA-NN has a different protein grouping algorithm from Spectronaut, we performed this analysis on the level of genes and peptide precursors. Employing similar grouping schemes at the gene level showed a high level of concordance, with 548 genes unique to Spectronaut and 208 unique to DIA-NN out of a total of 7668 identified genes for both (supplemental Fig. S11*B*). For the total of 128,002 identified peptide precursors, the discrepancy was somewhat larger, with 28% unique identifications for Spectronaut and 5% for DIA-NN (supplemental Fig. S11*C*). Overall, based on these proteome results, we conclude that the gains achieved by py_diAID are independent of the DIA analysis software used.

### Rapid Phosphoproteomics With Optimal Isolation Window Design

Phosphorylation, one of the most prevalent and most studied PTM, refers to the addition of a phosphoryl group—usually on serine, threonine, or tyrosine amino acid residues. This introduces a mass and IM shift on the modified peptides, indicating that analysis of phosphopeptides can benefit from the additional IM dimension in PASEF (44, 45). To date, dia-PASEF has not been explored in a large-scale study of the phosphoproteome or any other post-translationally modified subproteome.

It is well known that the IM dimension separates peptides in clouds primarily reflecting their charge status. In the timsTOF case, Figure 5*A* depicts dense clouds containing doubly, triply, and quadruply charged peptide ions (46). In the case of phospho-enriched samples, projecting the distribution of phosphorylated peptides into the *m/z* and IM space revealed a substantial shift of ion cloud to higher *m/z* values and higher IM values because of the 80 Da increase in their mass, higher charge states, and conformational changes upon phosphorylation (Fig. 5*B*). These observations suggest that dia-PASEF methods need to be tailored for phosphoproteomics. To this end, we first generated an in-depth phospholibrary from EGF-stimulated HeLa cells that were separated into 76 fractions and then enriched for phosphorylated peptides. These enriched fractions were measured with the 60 SPD method, DDA-PASEF in little more than 1 day. We analyzed the results both by FragPipe combined with DIA-NN and by Spectronaut 16 (see the Experimental Procedures section). This generated an in-depth library of 187,730 modified or unmodified peptides, 123,133 phosphopeptides, and 107,154 phosphosites for DIA-NN. Spectronaut 16 obtained very similar results (194,309 modified or unmodified peptides, 132,270 phosphopeptides, and 114,158 phosphosites). The overlap between phosphopeptides was 83% based on the sequence without considering the modification localizations (supplemental Fig. S14*A*).

When we simulated the coverage of the original dia-PASEF method for the 21 min gradient (6), we found that it only reached a coverage of 34% of phosphopeptide ions in our deep phospholibrary, in contrast to the 81% achieved for unmodified peptides (Fig. 5*C*). Therefore, we used our phospholibrary as input for py_diAID to obtain a dia-PASEF method tailored for phosphoproteomics. This resulted in a theoretical coverage of 93% of all doubly charged and 92% of all triply charged phosphopeptide ions (Fig. 5*D*).

We next utilized this optimal dia-PASEF phosphomethod to measure the samples containing phosphorylated peptides enriched from 100 μg digest of EGF-stimulated HeLa cells. We first analyzed the resulting files with DIA-NN against our deep phospholibrary. In agreement with our simulations, the original dia-PASEF method identified 8199 phosphosites and 13,485 phosphopeptides, whereas the optimal method detected 28% more phosphosites (10,510) and 43% more phosphorylated peptides (19,258) (Fig. 5*E*). The STY ratios of the identified phosphopeptides were similar for both methods, and the optimal method quantified 15,817 peptides modified on a serine, 3552 modified on a threonine, and 553 modified on a tyrosine (supplemental Fig. S12*A*). To illustrate this further, we mapped the experimentally acquired phosphopeptides to the EGFR sequence essential for transmitting the EGF signal using AlphaMap (47). This revealed that the optimal dia-PASEF phosphomethod doubled the number of detected phosphosites to a total of 14 (Fig. 5*F*).

The intensities of the phosphopeptides detected in our deep FragPipe phospholibrary in DDA–PASEF mode and 76 fractions span almost seven orders of magnitude (supplemental Fig. S13*A*). When searching single dia-PASEF phosphoruns against our phospholibrary using DIA-NN, we found that single short gradients covered 21% of the phosphopeptide sequences, ranging from 12% in the most abundant quintile to 0.3% in the least abundant one (supplemental Fig. S13*A*). Apart from the statistical analysis, the AlphaViz package (48), based on AlphaTims (49), allows visualization of any phosphopeptides of interest. This is shown for the phosphopeptide ELVEPLT[Phospho (STY)]PSGEAPNQALLR on EGFR, where the distinct precursor and fragment peaks are clearly visible in the RT dimension and even more important in the RT–IM plane, supporting the DIA-NN assignment (supplemental Fig. S13, *B* and *C*).

Next, we analyzed the same single-run phospho dataset with Spectronaut. To our surprise—especially given the comparable results at the proteome level—Spectronaut drastically increased the number of identified phosphosites to 28,980 (supplemental Fig. S14*B*). This was even more pronounced for identified phosphopeptides (72,216, supplemental Fig. S14*D*). Accordingly, the common overlap of phosphosites was only 26% (supplemental Fig. S14*B*) and 38% for the sequence of phosphopeptides without taking into account site localization (supplemental Fig. S14*C*).

We do not know the origin of this large discrepancy, but we encourage the providers of these software packages to resolve this, especially as the code is not available for inspection. In the context of our study, we decided to continue with the more extensive Spectronaut results, as they appeared to still correctly represent the regulation in the EGFR signaling experiment described later.

### In-Depth Phosphoproteomics Analysis of the EGF-Signaling Pathway

To benchmark our optimal dia-PASEF workflow, we chose the well-studied EGF signaling pathway in HeLa cells. The binding of EGF to the EGFR results in the activation of downstream kinases, which phosphorylate a repertoire of numerous substrates, regulating diverse cellular processes (50). We aimed to quantitatively and accurately measure the differential phosphorylation of proteins involved in this signaling pathway using our rapid and sensitive method. To this end, EGF-treated and control samples were collected in three biological replicates, digested into peptides, and enriched for phosphorylated peptides (see the Experimental Procedures section). Subsequently, we measured the enriched phosphopeptides with dia-PASEF in 21 min and searched the deep phosphopeptide library that we already employed for the method optimization described previously with Spectronaut 16.

**Fig. 6:**
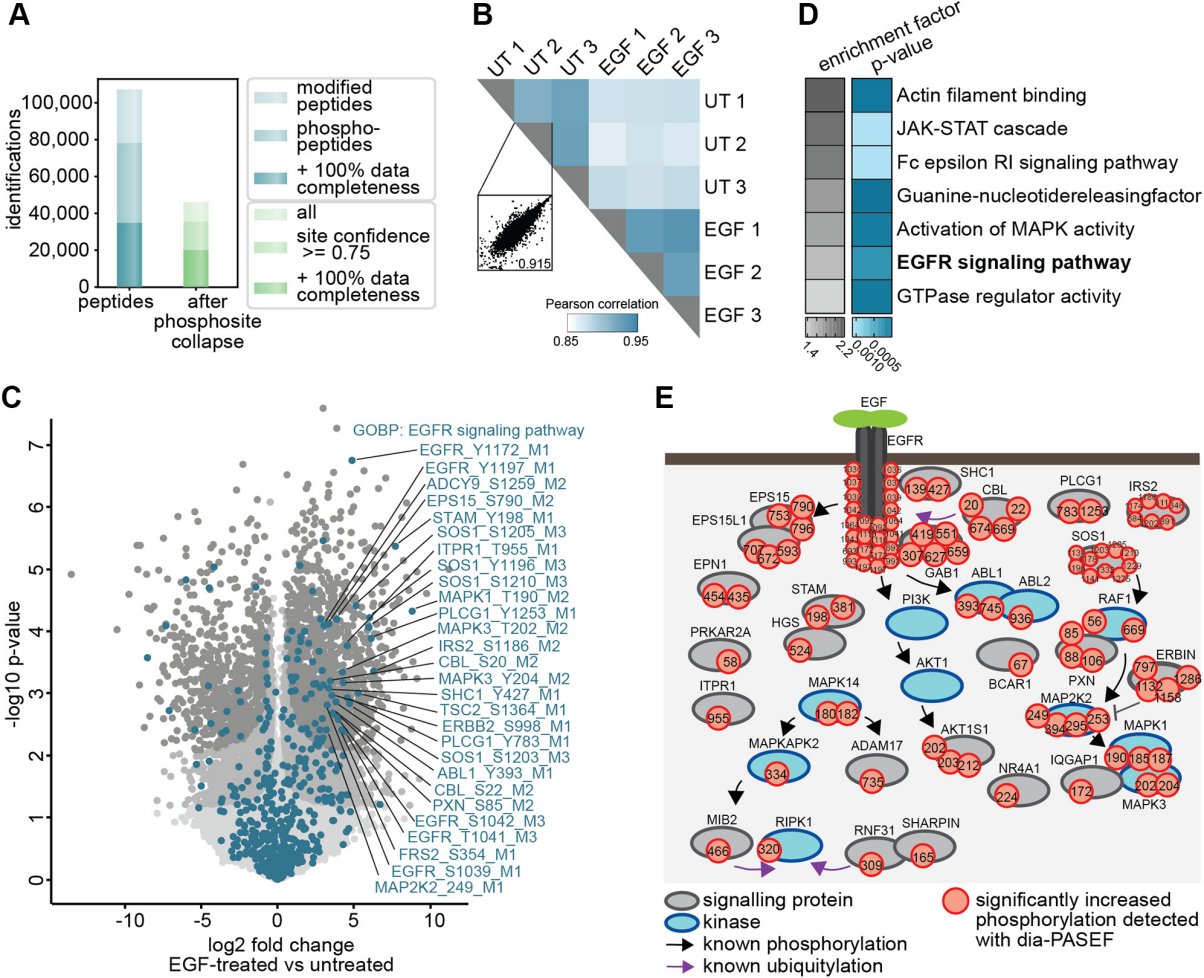
**The dia-PASEF workflow allows the robust detection of characteristic EGF signaling events.***A*, numbers of all identified phosphopeptides and phosphosites before and after filtering for localization probability and data completeness. *B*, phosphoproteome Pearson correlation matrix. Scatter plot shows the correlation of replicates within a condition. *C*, volcano plot of phosphosites regulated upon 15 min of EGF treatment in HeLa cells *versus* untreated cells. (Two-sided Student’s *t* test, FDR <0.01 = *gray*, FDR <0.05 = *dark gray*). Protein’s part of the GOBP term “EGFR signaling pathway” are highlighted in *turquoise*. *D*, Fisher’s exact test of proteins with significantly increased phosphosites upon EGF treatment (*p* < 0.002). Enrichment annotations are GOBP, GOMF, and KEGG. *E*, scheme of significantly upregulated phosphosites that were detected in this study and are part of the GOBP term “EGFR signaling pathway” and/or changed significantly upon EGF stimulation (FDR < 0.05). DIA, data-independent acquisition; EGF, epidermal growth factor; EGFR, EGF receptor; FDR, false discovery rate; GOBP, Gene Ontology Biological Process; GOMF, Gene Ontology Molecular Function; KEGG, Kyoto Encyclopedia of Genes and Genomes; PASEF, parallel accumulation–serial fragmentation.

With our workflow, we quantified 46,136 phosphorylation sites on 4300 proteins. Of these, 35,537 sites were identified with a high-site localization probability (75%, class I sites (51)) and 20,001 were quantified in all replicates of at least one experimental condition (Fig. 6*A*). Demonstrating the phosphoproteome depth, 62,057 phosphopeptides were reported by Spectronaut to have a modification on a serine, 19,513 on a threonine, and 2788 on a tyrosine (supplemental Fig. S12*B*). The dia-PASEF workflow allowed high reproducible quantification demonstrated by a median Pearson coefficient above 0.92 for replicates within conditions (Fig. 6*B*). Remarkably, a full 26% (5200, 5% FDR) and 10.5% (2117, 1% FDR) of phosphorylation sites were significantly modulated upon EGF treatment (Fig. 6*C*).

As expected, Gene Ontology enrichment analysis revealed strong overrepresentation of proteins involved in the EGFR signaling pathway (Gene Ontology Biological Process [GOBP]) and related pathways among the significantly EGF-upregulated phosphoproteins (Fig. 6*D*). Most are known to be critical for intact EGF signaling. For example, we detected phosphorylation of T693, Y1110, Y1172, and Y1197 on the receptor EGFR itself, Y427 on the adaptor protein Src homology 2 domain–containing-transforming protein C1 (SHC1), Y659 on growth factor receptor bound protein 2–associated protein 1 (GAB1), and on the downstream kinases mitogen-activated protein kinase 2 (MAP2K2) (T394) and mitogen-activated protein kinase 1 and 3 (MAPK1/3) (T185/Y187, T202/Y204) (52) (Fig. 6, *C* and *E*). These phosphosites are typically used to examine EGF signaling with classical methods such as immunoblotting or with targeted MS (9, 10, 53). These approaches, however, only allow relatively low-throughput analyses, which require dedicated assay development procedures or the generation of phosphospecific antibodies. In contrast, by combining the automated phosphoenrichment on the BRAVO platform with the robust Evosep and timsTOF setup, our approach achieves 60 SPD. This allows us to track and accurately quantify the induction of more than 60 phosphorylation events on proteins critical for EGF signaling (part of GOBP–EGFR signaling pathway) within a single 21-min run (supplemental Fig. S15). Importantly, besides the phosphorylations of the classical EGF signaling members, many other signaling events that, for example, result from signaling crosstalk downstream of the EGFR can also be detected, including S897 of the ephrin type-A receptor 2 (EPHA2), S339 of the C-X-C motif chemokine receptor 4 (CXCR4), and T701 of Erb-B2 receptor tyrosine kinase 2 (ERBB2) (supplemental Fig. S15).

To identify functionally important phosphorylation events not directly linked to EGF signaling, we matched the functionality prediction score developed by Beltrao *et al.* (54) to the upregulated phosphorylation events. We identified 659 phosphosites with a high functional score of >0.5 to be significantly upregulated, which are not part of the GOBP term “EGFR signaling pathway” (FDR < 0.05) (supplemental Data 1). These include EGF-induced phosphorylation of E3 ligases like Mindbomb homolog 2 (MIB2) (S309) and members of the linear ubiquitin chain assembly complex ring finger protein 31 (RNF31) (S466) and sharpin (S165), which are most frequently studied in the context of tumor necrosis factor signaling (supplemental Fig. S15) (55, 56, 57, 58). Similarly, phosphorylation of receptor interacting serine/threonine protein kinase 1 (RIPK1) on S320, which prevents tumor necrosis factor–induced cell death, was also increased upon EGF signaling (supplemental Fig. S15) (59, 60). This phosphorylation is mediated by MAP kinase–activated protein kinase 2 (MAPKAPK2), which is activated upon EGF stimulation demonstrated by its increased phosphorylation at T334. These are just some examples of functional candidates whose role in EGF signaling has still to be determined.

Together, this EGF study demonstrates the quantitative capabilities of the dia-PASEF-based phosphoproteomics workflow. We conclude that efficient analysis of ions separated in the IM and *m/z* space enables the investigation of signaling pathways with high sensitivity in a high-throughput manner.

## Discussion

The optimal placement of dia-PASEF windows in the two-dimensional *m/z* and IM space is not trivial. We here developed py_diAID, which is available on GitHub at MannLabs and is installable as a Python module with a command line interface or as a graphical user interface on Windows, Mac, and Linux. It adjusts the isolation window width to the precursor density and optimally positions the isolation design in the *m/z*–IM space. This leads to near-complete theoretical precursor coverage for proteomics. Compared with the original dia-PASEF method (6), the gains for phosphorylated precursors are especially striking (34% *versus* 93%).

MS-based proteomics is a rapidly developing technology. For perspective, to cover 10 thousand proteins, we had to measure the samples for 12 days with 4-h gradients 10 years ago (61). Here, we coupled a robust high-throughput LC system to the TIMS-qTOF instrument employing the rapid sampling speed of a TOF analyzer. It offers short gradients and also low overhead time, enhancing the overall throughput capabilities (28). With this, we generated in-depth project-specific libraries of 9461 proteins in only 13% of the previous measurement time. Furthermore, once the libraries are ready, subsequent proteome characterization using py_diAID-generated methods happens in only 44 min to a depth of 7700 proteins (less than 1% of the measurement time necessary 10 years ago). Our workflow is also twice as fast as currently employed high-throughput screening strategies for cancer proteomics, while achieving greater proteome depth on cell lysate (62, 63, 64).

So far, there have been only a few reports of the timsTOF principle on phosphoproteomics (29). Here, we show that this instrument is capable of in-depth phosphoproteomics with very high sensitivity. Specifically, we identified 35,000 phosphosites in only 21 min in triplicates from 100 μg EGF-stimulated HeLa cell digests. Our workflow opens up the possibility to measure multiple pathways in a short time. We demonstrated that quantitatively analyzing the regulated phosphoproteome covers the well-studied EGF signaling pathway together with auxiliary pathways. Interestingly, our workflow is even faster than selected reaction monitoring employed as a targeted screening method for assessing the activation of signaling pathways (9). However, our method is generic to any pathway and applicable in principle to the entire phosphoproteome.

In the current implementation, the dia-PASEF windows are adjusted based on empirical data before the acquisition. These adjustments could also be implemented in real time based on the precursor density achieving an acquisition design optimized to the individual time points of an entire gradient. Furthermore, we employed in-depth libraries. While they can be generated quickly, current developments of *in silico*-generated DIA libraries or direct DIA methods may soon obviate the need for this step. Likewise, we expect that py_diAID will perform similarly for other PTMs.

## Data Availability

All dia-PASEF parameter files required for the acquisition, MS raw files corresponding to the spectral libraries and single-run experiments, and output information from DIA-NN, Spectronaut, and FragPipe have been deposited with the ProteomeXchange Consortium *via* the PRIDE partner (65) repository with the dataset identifier PXD034128. Supplemental data 2 is a roadmap linking the raw files. *Homo sapiens* (taxon identifier: 9606) proteome databases were downloaded from https://www.uniprot.org. py_diAID is a fully open-source package, and the code is freely available under the Apache 2.0 license at https://github.com/MannLabs/pydiAID.

## Supplemental data

This article contains supplemental data (48, 49).

## Conflict of interest

M. M. is an indirect investor in Evosep Biosystems. All other authors declare no competing interests.
